# Prediction of 30-day in-hospital mortality in older UGIB patients using a simplified risk score and comparison with AIMS65 score

**DOI:** 10.1186/s12877-024-04971-w

**Published:** 2024-06-20

**Authors:** Zaiyao Xue, Hebin Che, Deyou Xie, Jiefeng Ren, Quanjin Si

**Affiliations:** 1 Medical School of Chinese PLA, Beijing, China; 2 Medical Big Data Research Center, Chinese PLA General Hospital, Beijing, China; 3 Beijing Research Center For Circulation Economy, Beijing, China; 4 The Third Healthcare Department, Second Medical Center of Chinese PLA General Hospital, National Clinical Research Center for Geriatric Diseases, Beijing, China

**Keywords:** Older adults, UGIB, In-hospital outcome prediction, AIMS65

## Abstract

**Background:**

Upper gastrointestinal bleeding (UGIB) in older patients is associated with substantial in-hospital morbidity and mortality. This study aimed to develop and validate a simplified risk score for predicting 30-day in-hospital mortality in this population.

**Methods:**

A retrospective analysis was conducted on data from 1899 UGIB patients aged ≥ 65 years admitted to a single medical center between January 2010 and December 2019. An additional cohort of 330 patients admitted from January 2020 to October 2021 was used for external validation. Variable selection was performed using five distinct methods, and models were generated using generalized linear models, random forest, support vector machine, and k-nearest neighbors approaches. The developed score, “ABCAP,” incorporated Albumin < 30 g/L, Blood Urea Nitrogen (BUN) > 7.5 mmol/L, Cancer presence, Altered mental status, and Pulse rate > 100/min, each assigned a score of 1. Internal and external validation procedures compared the ABCAP score with the AIMS65 score.

**Results:**

In internal validation, the ABCAP score demonstrated robust predictive capability with an area under the curve (AUC) of 0.878 (95% CI: 0.824–0.932), which was significantly better than the AIMS65 score (AUC: 0.827, 95% CI: 0.751–0.904), as revealed by the DeLong test (*p* = 0.048). External validation of the ABCAP score resulted in an AUC of 0.799 (95% CI: 0.709–0.889), while the AIMS65 score yielded an AUC of 0.743 (95% CI: 0.647–0.838), with no significant difference between the two scores based on the DeLong test (*p* = 0.16). However, the ABCAP score at the 3–5 score level demonstrated superior performance in identifying high-risk patients compared to the AIMS65 score. This score exhibited consistent predictive accuracy across variceal and non-variceal UGIB subgroups.

**Conclusions:**

The ABCAP score incorporates easily obtained clinical variables and demonstrates promising predictive ability for 30-day in-hospital mortality in older UGIB patients. It allows effective mortality risk stratification and showed slightly better performance than the AIMS65 score. Further cohort validation is required to confirm generalizability.

**Supplementary Information:**

The online version contains supplementary material available at 10.1186/s12877-024-04971-w.

## Background

Upper gastrointestinal bleeding (UGIB) represents a significant clinical challenge, marked by its high prevalence and substantial impact on public health systems globally. In the United Kingdom, the annual incidence of UGIB ranges from 103 to 172 cases per 100,000 adults, accompanied by a mortality rate of 8–14% [1]. The situation is similarly dire in the United States, where UGIB accounts for over 800,000 emergency department visits annually, with about half of these cases necessitating hospitalization [2]. In China, the UGIB-specific death rate is estimated to be between 4–14% [4], highlighting the universal burden of this condition.

Evidence suggests that effective prediction tools can significantly impact patient outcomes by facilitating early intervention and appropriate care management [8].Tools such as the Glasgow-Blatchford score [5], the Rockall score [3], and the AIMS65 score [6] have been pivotal in predicting in-hospital mortality and guiding clinical management. These instruments aid in patient stratification, potentially diminishing hospital stays and optimizing resource use [9]. Recent studies highlight that urgent endoscopic procedures guided by high AIMS65 scores may contribute to reduced hospitalization periods for patients with nonvariceal upper gastrointestinal bleeding [10].

However, the effectiveness of current prediction tools for managing conditions in the older adult population is marked by uncertainty. This group’s diverse physiological reserves and comorbid conditions challenge the applicability of generalized prediction models. Research into predictive tools for various diseases underscores this issue; for instance, existing cardiovascular risk models [11] and scores for acute respiratory infections [12] have shown limitations when applied to older adults, signaling the urgent need for adaptations that consider age-specific factors. This highlights the essential demand for developing more specialized models tailored to the unique needs of these populations. Specifically for older UGIB patients, evaluating whether existing prediction tools remain effective and determining the necessity for a tailored tool warrant further investigation.

As the global population ages rapidly, it becomes imperative to focus on older adults and reevaluate existing scoring systems, especially in contexts like China’s, where demographic shifts are particularly pronounced [7]. This study aims to develop a robust predictive model for estimating the 30-day in-hospital all-cause mortality among older patients with UGIB prior to endoscopic evaluation. We also plan to compare the predictive performance of this new score against the widely utilized AIMS65 score. Through these research endeavors, we aspire to refine risk stratification techniques, bolster clinical decision-making, and ultimately improve the management and outcomes of older patients with UGIB.

## Methods

### Participants

The source of participants for this study is the Older Diseases Dataset, a well-established and continuously updated research dataset comprising individuals aged over 60 years. The dataset is derived from the Electrical Health Record of the First Medical Center of the Chinese People’s Liberation Army General Hospital (PLAGH). The development dataset encompasses patients admitted between January 2010 and December 2019, as per the available version before 2022. Furthermore, an external validation dataset was employed to assess the predictive score’s performance beyond its development dataset. This validation dataset comprises patients admitted between January 2020 and October 2021.

Inclusion Criteria:

- Age at admission greater than 65 years.
- Admission diagnosis of UGIB (blood loss from a gastrointestinal source above the ligament of Treitz), including diagnoses documented by physicians, admission records, and corresponding International Classification of Diseases 10th codes (ICD-10).
- Typical symptoms described in patient complaints and medical history, such as “hematemesis (vomiting of fresh blood),” “coffee-ground” emesis (vomiting of dark altered blood), and/or melena.
- Identification by a gastroenterologist.
- For patients with multiple admissions, only data from the earliest hospitalization were considered.

Exclusion Criteria:

- Unclassified gastrointestinal bleeding.
- Nonbleeding periods, such as old bleeding episodes or bleeding history.
- Cases with a significant (> 50%) amount of missing laboratory results.

### Data collection

#### ICD-10 codes for UGIB

The ICD-10 codes of UGIB: see the supplementary document.

#### Laboratory test indicators

Priority was given to data collected within 24 h of admission, including hemoglobin (HGB, g/dL), platelet count (PLT, 10^3/µL), international normalized ratio (INR), Albumin (Albumin, g/L), blood urea nitrogen (BUN, mmol/L), creatinine(CR,µmol/L) and estimated glomerular filtration rate(eGFR), which was calculated using the following formula: eGFR(ml/(min*1.73m^*2*^*)) = 186×(Scr)*^*-1.154*^*×(Age)*^*-0.203*^ × (0.742Female)*1.233. Several other critical indicators, such as white cell counts and prothrombin time, were excluded from the analysis due to issues related to multicollinearity during preliminary work. Indicators with missing values exceeding 20% are not collected.

#### Vital signs, checkup, and mental status

Vital signs, including systolic blood pressure (SBP, mmHg), diastolic blood pressure (DBP, mmHg) and pulse (beats per minute) were recorded. Body mass index (BMI) was calculated using the following formula: *BMI = weight (kg) / height*^*2*^*(m*^*2*^*)*. Altered mental status was defined as a Glasgow Coma Scale score of less than 14 or a physician-charted designation of “disoriented,” “lethargy,” “stupor,” or “coma.”

#### Charlson comorbidity index and comorbidities

The collection of comorbidities for the Charlson Comorbidity Index (CCI) involved the utilization of ICD-10 codes, with subsequent calculation of the CCI score for each patient by summing the assigned weights of the respective comorbidities (as detailed in the supplementary document). CCI serves as an extensively utilized tool for prognosticating 10-year survival rates among patients grappling with multiple comorbid conditions [13, 14]. This index attributes specific weights to diverse comorbidities in accordance with their individual impact on prognosis. In our study, the enumeration of CCI components was instrumental in depicting the intricate landscape of health challenges faced by the older population.

The spectrum of collected comorbidities encompassed an array of conditions, including coronary heart disease (CAD), congestive heart failure (CHF), peripheral vascular disease (PAD), cerebrovascular disease (CVD), chronic obstructive pulmonary disease (COPD), moderate to severe kidney disease (Kidney Diseases), and liver disease (Liver Diseases). Additionally, cancers, both metastatic and nonmetastatic, as well as other conditions featured in the CCI were comprehensively incorporated. Furthermore, common geriatric comorbidities such as hypertension (HTN) and atrial fibrillation (AF) were meticulously recorded in the dataset, contributing to the comprehensive portrayal of the patients’ health status.

#### Endoscope and outcome

Endoscopy records during hospitalization were collected. The primary outcome of interest was defined as any death occurring within 30 days of hospitalization with UGIB. Additionally, we also collected data on the length of hospital stay (days, LOS).

### Quality control

Two independently trained investigators analyzed and collected data from electronic medical records. In case of conflicts, higher-level personnel make the final determination.

### Statistical, data handling, training and evaluation methods

#### Statistical methods

All statistical analyses were conducted using R (R Version:4.2.3.R Core Team (2023). R: A language and environment for statistical computing. R Foundation for Statistical Computing, Vienna, Austria. URL https://www.R-project.org/). Normally distributed variables are presented as the mean ± standard deviation, while nonnormally distributed variables are described as the median (interquartile range). Group comparisons for normally distributed variables employed t tests, whereas the Kruskal-Wallis test was applied for nonnormally distributed variables. Categorical variables were compared using the chi-square test or Fisher’s exact test. To assess the association of each variable with the outcome, univariable regression was used.

#### Training and validation dataset

The Development dataset was further divided into a 70% training subset, used for variable selection and model training, and a 30% internal validation subset. The external validation dataset was employed for independent model evaluation and to ensure the robustness of the model performance assessment. The random partitioning of data into these subsets was carried out utilizing the ‘createDataPartition’ function available within the ‘caret’ package.

#### Missing value handling

In our study, the missing values, mainly attributed to the lack of testing within a specific time window, can be classified as “Missing Completely at Random” (MCAR). In MCAR scenarios, the absence of data is assumed to be unbiased and unlikely to systematically affect the outcomes, simplifying the process of imputation and analysis. To address these missing values within the development dataset, we employed appropriate procedures.

To ensure the integrity and reliability of the results, variables with missing data exceeding 20% were excluded from the analysis. Additionally, individual cases with missing values surpassing 50% in laboratory indicators were excluded during the data screening process. For instances where missing values were less than 20%, we utilized the ‘missForest’ function from the ‘missForest’ package in R for imputation. To enhance the reliability of the imputed data, we repeated the imputation process five times, and a statistical test was conducted to compare the imputed data with the original dataset. This comparison confirmed the absence of significant discrepancies between the imputed data and the original dataset. Detailed information can be found in the supplementary document.

#### Variables selection methods

To enhance the clinical applicability and interpretability of the predictive model, continuous variables were transformed into categorical variables using both general standard cutoff values and specific cutoff values employed in the First Medical Center of PLAGH. To identify the most relevant predictors and reduce dimensionality, we employed five variable selection methods: Stepwise by Akaike Information Criterion (‘StepAIC’, ‘MASS’ package), Least Absolute Shrinkage and Selection Operator (‘LASSO’, ‘glmnet’ package), Elastic net (‘ENT’, ‘glmnet’ package), Best subset (‘BestSub’, ‘leaps’ package), and Recursive Feature Elimination (‘RFE’, ‘caret’ package). All selection methods were applied to the five training iterations, and the resulting variable selection outcomes from each method were combined. In cases where the number of selected variables exceeded 10, we included the top 10 variables that appeared most frequently in the selection results.

#### Model training methods and evaluation in internal and external evaluation

In our training dataset, we applied four distinct model training techniques using the ‘train’ function from the ‘caret’ package. These methods encompass Generalized Linear Models (GLM), k-Nearest Neighbors (KNN), Support Vector Machine (SVM), and Random Forest (RF). Each was thoughtfully chosen to harness its specific advantages in capturing the complex relationships between predictor variables and the outcome variable.

To ensure robust model assessment and address the inherent limitations of our sample size, we employed a Repeated k-fold Cross-Validation approach, utilizing the ‘trainControl’ function from the ‘caret’ package. This cross-validation method was executed five times, each time with 20 folds, enabling us to obtain more stable and reliable estimates of our models’ performance while mitigating the risk of overfitting.

To train the models, we performed the training process on the five training datasets using the variable selection subsets obtained from the feature selection process. Subsequently, the trained models were evaluated on the corresponding five internal validation datasets, enabling a comprehensive assessment of their performance across different data partitions. During the evaluation process, various performance metrics, including specificity, sensitivity, accuracy, F1 score, and area under the curve (AUC), were calculated for each iteration on the internal validation. To provide a more reliable estimate of the models’ overall performance, the mean value of each performance metric across the five internal validation iterations was calculated.

Once the optimal model was identified and the scoring system was established, we employed AUC as the metric to validate its performance. This validation encompassed a comparison of the model’s performance with the established AIMS65 score, across both internal and external validation phases.

## Results

### Characteristics of participants in the development dataset

**Fig. 1:**
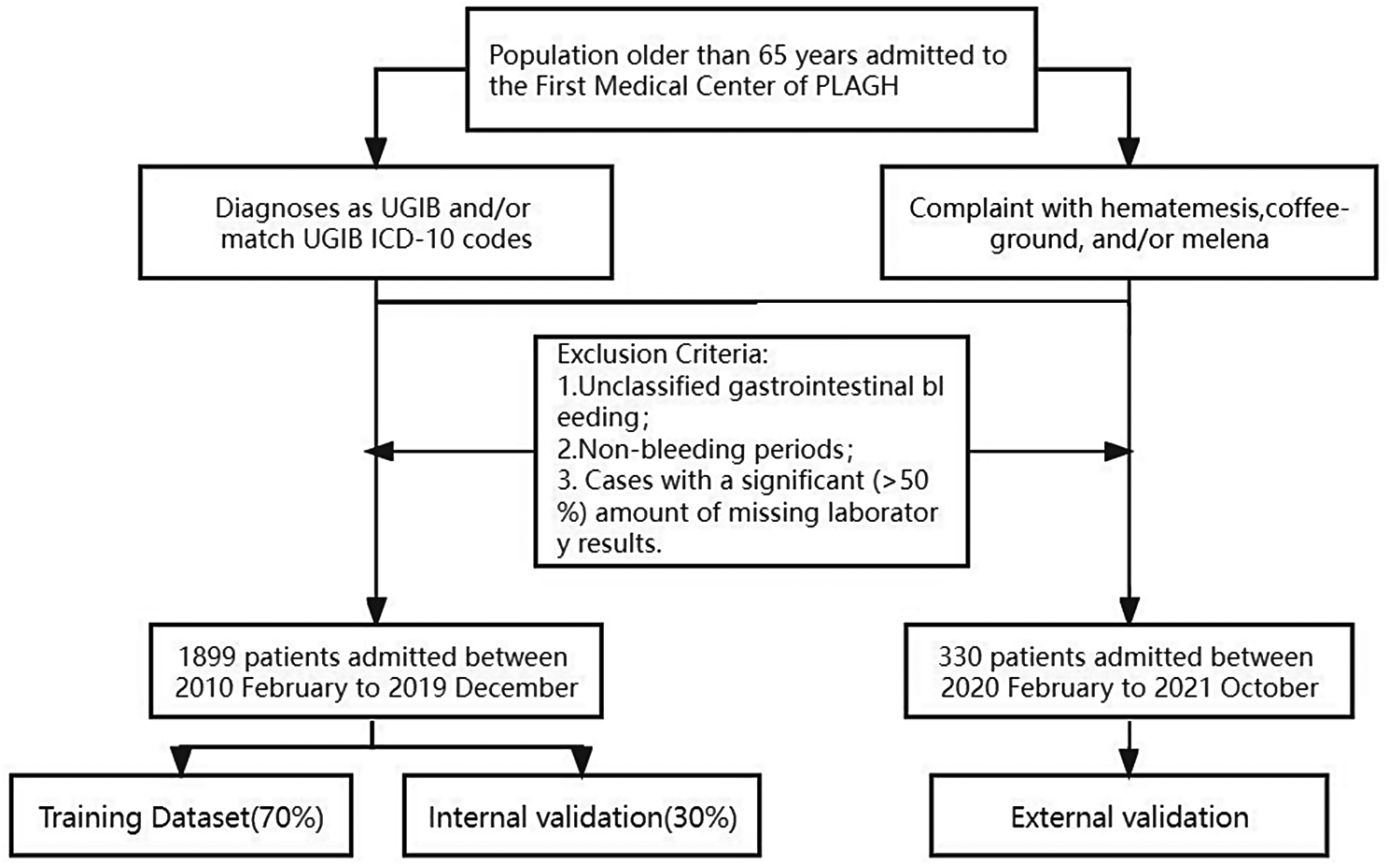
Flow diagram of participants in study

A total of 1899 distinct patients diagnosed with UGIB were included in the development dataset, and subsequently divided into training and internal validation subsets. To ensure rigor, the inclusion and exclusion criteria detailed in the Methods section were meticulously applied. Additionally, an external validation dataset consisting of 330 patients was defined, maintaining alignment with the established criteria. A flow diagram illustrating the participant selection process is presented in Fig. 1. The cohort comprised patients with a median age of 72 years, ranging from 65 to 102 years. During the 30-day follow-up period, a total of 97 patients experienced mortality associated with the condition, with an additional 21 patients passing away after the 30-day period.

Within the development dataset, a comparative analysis was conducted to investigate potential differences between patients who died within 30 days and those who survived. Demographic characteristics, comorbidities, and laboratory results were compared and summarized in Table 1.

In Table 1, it is noteworthy that the “Alive” group exhibits a notably higher ratio of in-hospital endoscopy, variceal cases, and alcohol use compared to the “Death” group, signifying a statistically significant association. Conversely, the “Death” group demonstrated an elevated mean age, increased CCI value, and a greater frequency of altered mental status. Additionally, the “Death” group shows a comparatively shorter LOS compared to the “Alive” group.

**Table 1:** Baseline characteristics of patients in the development dataset

Characteristic^1^	Alive at 30 d, *N* = 1,802	Death within 30 d, *N* = 97	*p*-value^2^	Univariable logistic regression	
				OR(95%CI)	*p*-value
AGE (years) Median (IQR^3^)	72 (68, 78)	78 (71, 83)	< 0.001	1.08(1.05,1.11)	<0.000
Female (*n*, %)	729 (40%)	37 (38%)	0.651	1.1(0.73,1.69)	0.651
In-hospital endoscope	1324(73%)	38(39%)	< 0.000	0.23(0.15,0.35)	<0.000
Altered mental status(*n*, %)	93 (5.5%)	34 (41%)	< 0.001	12.13(7.42,19.69)	< 0.000
Smoke history(*n*, %)	421 (25%)	23 (28%)	0.532	1.17(0.7,1.89)	0.533
Alcohol use(*n*, %)	453(25%)	15(15%)	0.031	0.54(0.3,0.93)	0.034
Comorbidities(*n*, %)					
Variceal	601 (33%)	21 (22%)	0.017	0.55(0.33,0.89)	0.018
Peptic ulcer	353 (20%)	21 (22%)	0.619	1.13(0.67,1.83)	0.619
ICH	33 (1.8%)	5 (5.2%)	0.041	2.91(0.98,7.01)	0.030
CAD	89 (4.9%)	12 (12%)	0.001	2.72(1.37,4.98)	0.002
Liver diseases	666 (37%)	36 (37%)	0.976	1.01(0.65,1.53)	0.976
Diabetes	405 (22%)	23 (24%)	0.777	1.07(0.65,1.71)	0.777
Cancer	313 (17%)	39 (40%)	< 0.001	3.2(2.08,4.87)	< 0.000
HTN	645 (36%)	49 (51%)	0.003	1.83(1.21,2.76)	0.004
AF	85 (4.7%)	5 (5.2%)	0.805	1.1(0.38,2.52)	0.843
CVD	236 (13%)	23 (24%)	0.003	2.06(1.24,3.31)	0.004
CHF	66 (3.7%)	14 (14%)	< 0.001	4.44(2.31,8.01)	< 0.000
COPD	26 (1.4%)	3 (3.1%)	0.182	2.18(0.51,6.34)	0.208
Kidney diseases	56 (3.1%)	13 (13%)	0.001	4.83(2.44,8.92)	< 0.000
CCI Median (IQR)	5 (4, 6)	7 (5, 9)	< 0.001	1.44(1.32,1.57)	< 0.000
LOS(days) Median (IQR)	14 (9, 21)	5 (2, 14)	< 0.001		
Physical & Lab Examination (IQR)					
BMI(kg/m^2^;)	23.4 (21.0, 25.8)	21.8 (19.5, 24.4)	< 0.001	0.9(0.84,0.96)	0.001
DBP(mmHg)	71 (65, 80)	65 (58, 72)	< 0.001	0.95(0.93,0.97)	< 0.000
SBP(mmHg)	130 (118, 140)	117 (100, 136)	< 0.001	0.97(0.96,0.98)	< 0.000
Pulse(per minute)	78 (72, 83)	87 (78, 104)	< 0.001	1.06(1.05,1.07)	< 0.000
PLT(10^9/L)	128 (46, 211)	89 (53, 145)	0.040	1(0.99,1)	0.049
HGB(g/L)	96 (80, 117)	85 (73, 105)	0.003	0.98(0.97,0.99)	0.001
Albumin(g/L)	34.9 (30.7, 39.0)	27.4 (22.0, 31.9)	< 0.001	0.83(0.79,0.86)	< 0.000
BUN(mmol/L)	5.5 (4.2, 7.6)	10.7 (6.9, 18.3)	< 0.001	1.12(1.09,1.15)	< 0.000
INR	1.14 (1.05, 1.28)	1.32 (1.20, 1.62)	< 0.001	2.09(1.43,3.04)	< 0.000
CR(µmol/L)	71 (59, 86)	81 (64, 125)	0.003	1.00(1.00,1.01)	<0.000
eGFR(ml/min/1.73 m²)	110 (91, 132)	95 (63, 133)	0.002	0.99(0.98,0.99)	0.001

^1^*n* (%)Before imputation

^2^Pearson’s Chi-squared test; Fisher’s exact test; Wilcoxon rank sum test

^3^IQR = Interquartile range

### Variables subset selection

Following the completion of imputation and dataset splitting, a comprehensive variable selection process was initiated, employing five distinct methods on the entire training dataset. The outcomes of these selection methods exhibited varying patterns, as shown in Table 2. A total of 15 variables were selected by five methods. The StepAIC, RFE and BestSub methods identified more than 10 different significant features, while the 10 most frequently occurring features are included in Table 2. LASSO and ENT have one relatively fixed result. INR emerged as a significant variable selected by four of the methods, while HF and Variceal were each chosen by three methods. Interestingly, SBP was exclusively chosen by two specific methods. Variables such as AGE, eGFR, HGB, ICH, Liver Diseases, and Peptic Ulcer were each selected by a single method.” Based on the consistent selections of important variables across the all methods, including “Albumin”, “BUN”, “Cancer”, “Altered Mental” and “Pulse”, we can create another subset called “ABCAP” that includes these five variables.

**Table 2:** Variables selection results by different methods in the training dataset

Variable	Categorical	StepAIC	LASSO	ENT	RFE	BestSub
AGE	75 years					✓
Albumin	30 g/L	✓	✓	✓	✓	✓
BUN	7.5 mmol/L	✓	✓	✓	✓	✓
Altered mental	Yes/No	✓	✓	✓	✓	✓
eGFR	< 30 ~ 90< (ml/min/1.73 m²)				✓	
CHF	Yes/No	✓		✓		✓
HGB	120 g/L(Male); 110 g/L(Female)	✓				
ICH	Yes/No	✓				
INR	1.5	✓		✓	✓	✓
Liver diseases	Yes/No				✓	
Peptic ulcer	Yes/No				✓	
Pulse	100/min	✓	✓	✓	✓	✓
SBP	90 mmHg		✓			✓
Cancer	Yes/No	✓	✓	✓	✓	✓
Variceal	Yes/No	✓			✓	✓
No.	15	10	6	7	10	10

### Performance of models combined with variable subsets in internal validation

The evaluations of various training methods combined with different feature selection results in internal validation are presented in Table 3. For instance, the combination of KNN + StepAIC means using the KNN training prediction model with the variable subset selected by StepAIC. All combinations consistently achieved accuracy, sensitivity, and F1 score levels slightly above 0.9.Combinations involving RF, KNN, and SVM models exhibited issues with correctly identifying negative instances, resulting in decreased overall AUC values, especially for SVM. The GLM models consistently outperformed the other methods. Among them, GLM + BestSub showed the best performance of all and achieved the highest AUC (0.89,95% CI: 0.831, 0.949), with other GLM combinations also showing promising results. The AUC for GLM + ABCAP was 0.879 (95% CI: 0.818, 0.939), slightly below the AUC of 0.888 for GLM + BestSub, which includes only five key variables.

**Table 3:** Model performance in internal validation

	Accuracy	Sensitivity	Specificity	F1 Score	AUC
KNN + StepAIC	0.951 ± 0.002	0.953 ± 0.002	0.71 ± 0.213	0.975 ± 0.001	0.872 (0.8, 0.943)
KNN + LASSO	0.953 ± 0.003	0.956 ± 0.001	0.69 ± 0.192	0.976 ± 0.001	0.813 (0.716, 0.909)
KNN + ENT	0.951 ± 0.001	0.955 ± 0.003	0.7 ± 0.184	0.975 ± 0.000	0.825 (0.736, 0.915)
KNN + BestSub	0.951 ± 0.002	0.953 ± 0.001	0.72 ± 0.189	0.975 ± 0.001	0.863 (0.782, 0.943)
KNN + RFE	0.951 ± 0.005	0.953 ± 0.004	NA ± NA	0.975 ± 0.002	0.833 (0.745, 0.921)
RF + StepAIC	0.949 ± 0.004	0.954 ± 0.003	0.659 ± 0.317	0.974 ± 0.002	0.8 (0.707, 0.893)
RF + LASSO	0.951 ± 0.002	0.959 ± 0.003	0.56 ± 0.034	0.975 ± 0.001	0.769 (0.676, 0.862)
RF + ENT	0.95 ± 0.002	0.953 ± 0.002	0.55 ± 0.17	0.974 ± 0.001	0.806 (0.717, 0.896)
RF + BestSub	0.951 ± 0.004	0.957 ± 0.001	0.578 ± 0.132	0.975 ± 0.002	0.808 (0.718, 0.897)
RF + RFE	0.953 ± 0.004	0.956 ± 0.002	0.745 ± 0.228	0.976 ± 0.002	0.84 (0.759, 0.92)
SVM + StepAIC	0.947 ± 0.002	0.953 ± 0.002	0.393 ± 0.068	0.973 ± 0.001	0.76 (0.66, 0.859)
SVM + LASSO	0.95 ± 0.003	0.956 ± 0.005	0.495 ± 0.113	0.974 ± 0.002	0.67 (0.552, 0.787)
SVM + ENT	0.948 ± 0.003	0.953 ± 0.002	0.438 ± 0.133	0.973 ± 0.002	0.665 (0.55, 0.779)
SVM + BestSub	0.949 ± 0.005	0.954 ± 0.004	0.544 ± 0.29	0.974 ± 0.002	0.711 (0.589, 0.832)
SVM + RFE	0.951 ± 0.004	0.955 ± 0.001	0.689 ± 0.289	0.975 ± 0.002	0.695 (0.57, 0.821)
GLM + StepAIC	0.956 ± 0.006	0.963 ± 0.006	0.65 ± 0.128	0.977 ± 0.003	0.884 (0.822, 0.946)
GLM + LASSO	0.955 ± 0.006	0.961 ± 0.004	0.675 ± 0.165	0.977 ± 0.003	0.88 (0.818, 0.943)
GLM + ENT	0.955 ± 0.006	0.962 ± 0.004	0.645 ± 0.157	0.977 ± 0.003	0.878 (0.813, 0.943)
GLM + BestSub	0.956 ± 0.008	0.963 ± 0.006	0.67 ± 0.173	0.977 ± 0.004	0.89 (0.831, 0.949)
GLM + RFE	0.956 ± 0.007	0.963 ± 0.004	0.669 ± 0.158	0.977 ± 0.003	0.882 (0.819, 0.945)
GLM + ABCAP	0.957 ± 0.007	0.962 ± 0.005	0.691 ± 0.157	0.978 ± 0.003	0.879 (0.818, 0.939)

**Fig. 2:**
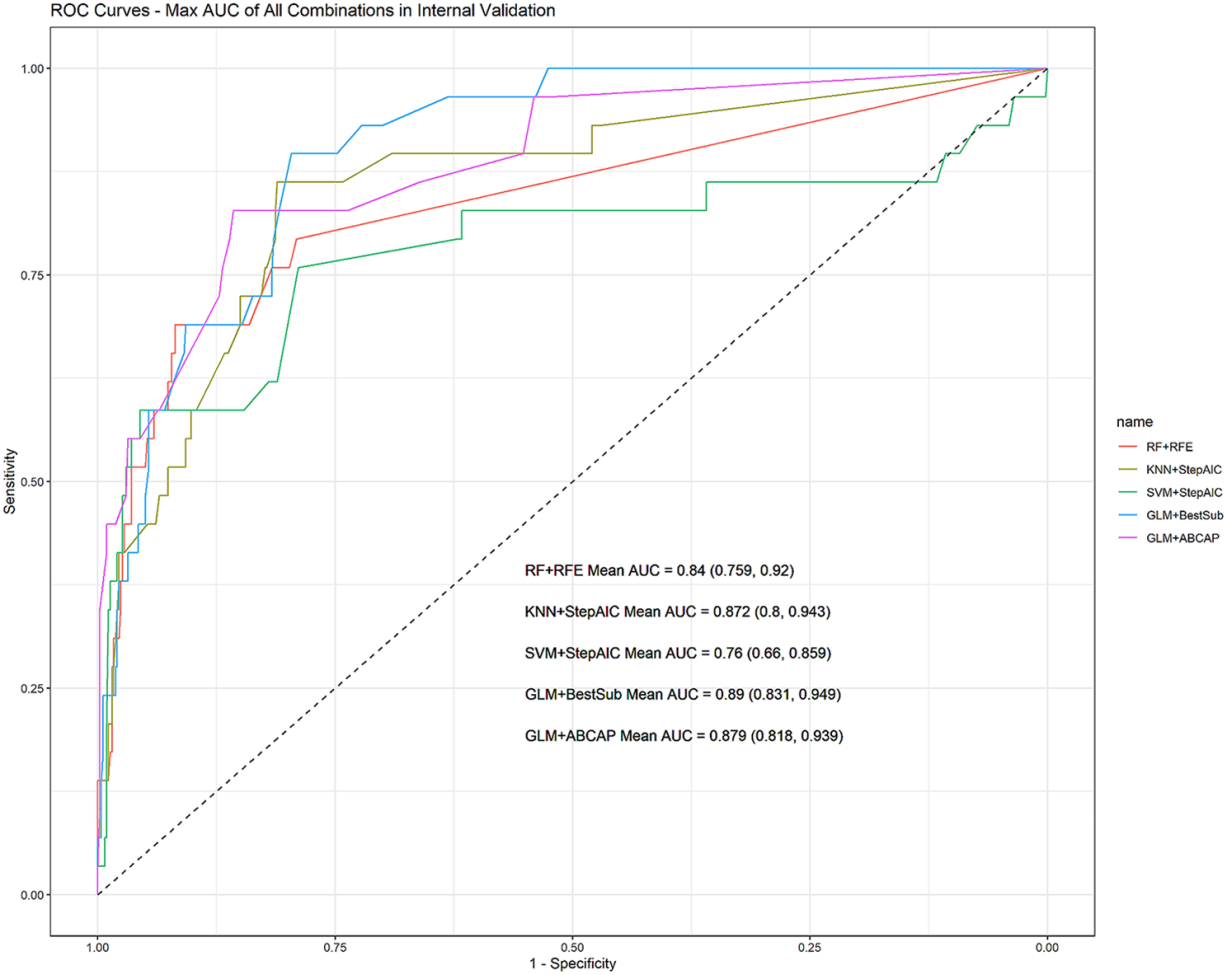
Max AUC of each training methods in internal validation

Figure 2 displays the receiver operating characteristic (ROC) curves for each combination with the highest AUC, including the GLM + ABCAP combination. While GLM + BestSub emerged as the top performer in terms of AUC, it’s essential to consider the balance between model complexity and performance. Notably, the GLM + ABCAP combination offers a more parsimonious model by utilizing only five variables, compared to the ten variables used by GLM + BestSub. This suggests that the GLM + ABCAP combination may be a more appropriate choice when seeking a simpler and more interpretable model, without a significant sacrifice in predictive performance.

### General linear models equation and ABCAP score

After carefully considering various factors, we chose the GLM + ABCAP combination as our final model. Consequently, the resulting GLM equation, trained using the training dataset, takes the form:

$$\begin{aligned}&\text{l}\text{o}\text{g}\left[\frac{\widehat{P\left(\text{O}\text{U}\text{T}\text{C}\text{O}\text{M}\text{E}=\text{D}\text{e}\text{a}\text{t}\text{h}\right)}}{1-\widehat{P\left(\text{O}\text{U}\text{T}\text{C}\text{O}\text{M}\text{E}=\text{D}\text{e}\text{a}\text{t}\text{h}\right)}}\right]\\&\quad=-3.71+1.60\left({\text{C}\text{a}\text{n}\text{c}\text{e}\text{r}}_{1}\right)+1.59\left(\text{A}\text{l}\text{t}\text{e}\text{r}\text{e}\text{d}{\text{M}\text{e}\text{n}\text{t}\text{a}\text{l}}_{1}\right)\\&\quad+1.40\left({\text{P}\text{u}\text{l}\text{s}\text{e}}_{2}\right)-1.39\left({\text{A}\text{l}\text{b}\text{u}\text{m}\text{i}\text{n}}_{2}\right)+1.35\left({\text{B}\text{U}\text{N}}_{2}\right)\end{aligned}$$

In the GLM equation, each variable is represented by a coefficient value, indicating its contribution to the log-odds of the outcome (death). A positive coefficient suggests a positive association with the outcome, while a negative coefficient suggests a negative association. By plugging in the respective values of the variables, the equation allows for the estimation of the probability of the outcome being death.

The equation and Table 4 reveal important associations between the variables and the outcome. The intercept term of -3.71 represents the log-odds of the outcome when all predictor variables are at their reference levels or baseline values. Among the five variables integrated into the model, Cancer, Altered Mental, Pulse (> 100/min), and BUN (> 7.5 mmol/L) exhibit positive coefficients, indicating an elevated risk of the outcome. Conversely, Albumin (≥ 30 g/L) has a negative coefficient, suggesting a protective effect on prognosis, while Albumin (< 30 g/L) has the opposite effect. The odds ratios further quantify these associations. Odds ratios exceeding 1 signify an increased risk, whereas odds ratios below 1 indicate a decreased risk.

**Table 4:** Generalized linear models with ABCAP variables in the training dataset

Variable	β-coefficient	OR	95%CI	*p*-value
Cancer	1.60	4.943	2.671–9.154	< 0.000
Altered mental status	1.59	5.276	2.679–10.394	< 0.001
Pulse>100/min	1.40	4.151	2.086–8.263	< 0.000
Albumin ≥ 30 g/L	-1.39	0.254	0.138–0.465	< 0.001
BUN>7.5mmol/L	1.35	3.894	2.044–7.422	< 0.001

The ABCAP score, derived from the GLM equation mentioned earlier, simplifies calculations by assigning one point to each of the five predictors. In parallel, the AIMS65 score—a validated scoring system for predicting in-hospital mortality in patients with UGIB—also incorporates five variables, with each variable assigned a score of 1 point [6].

Table 5 compares variables in both scoring systems used to assess severity and predict in-hospital mortality for patients with UGIB. The ABCAP score includes Cancer, Altered Mental Status, Pulse > 100/min, Albumin < 30 g/L, and BUN > 7.5mmol/L. In contrast, the AIMS65 score involves Albumin < 30 g/L, INR > 1.5, Altered Mental Status, SBP ≤ 90mmHg, and Age ≥ 65 years. Both the ABCAP and AIMS65 scores share commonalities by incorporating Albumin and Altered Mental Status as indicators of disease severity. Notably, the ABCAP score does not consider Age, INR, and SBP as scoring criteria, distinguishing it from the AIMS65 score.

**Table 5:** Features and points of the ABCAP score and AIMS65 score

	Variable	Parameter	Score
ABCAP score	Albumin	< 30 g/L	1
	BUN	>7.5 mmol/L	1
	Cancer	Yes/No	1
	Altered mental	Yes/No	1
	Pulse	>100/min	1
AIMS65 score	Albumin	< 30 g/L	1
	INR	> 1.5	1
	Mental status	Altered	1
	SBP	≤ 90 mmHg	1
	Age	≥ 65 years	1

### Comparison of ABCAP score with AIMS65 score in internal and external validation

**Fig. 3:**
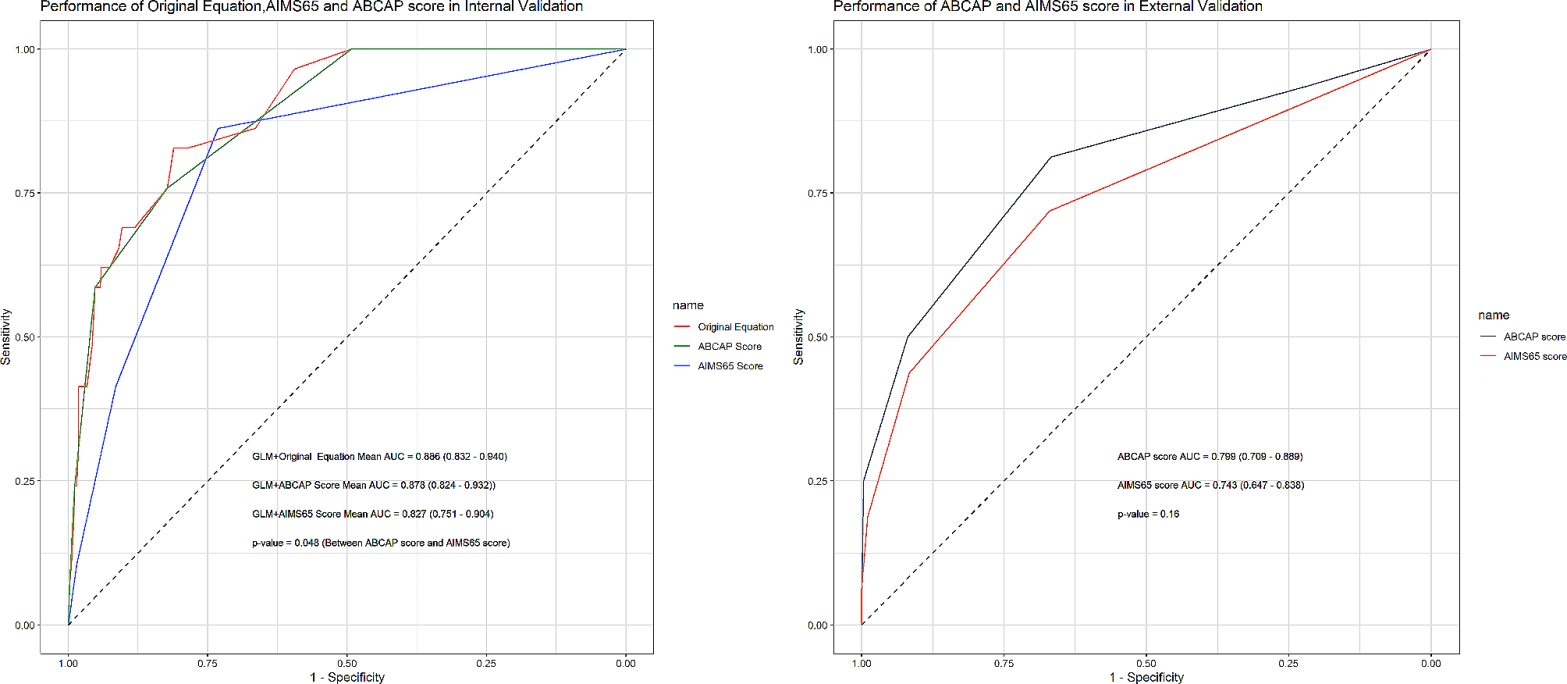
AIMS65 score, ABCAP score and original equation performance in internal and external validation

Table 6 presents the characteristics of the external validation dataset. All variables included in both scoring systems exhibited significant differences between the groups of patients who survived and those who did not. Figure 3 shows the ROC curves of the original equation, AIMS65 and ABCAP score in both internal and external validations. In the internal validation, the original equation achieves an AUC of 0.886 (95% CI: 0.832–0.940), the ABCAP score attained an AUC of 0.878 (95% CI: 0.824–0.932), and the AIMS65 score demonstrated an AUC of 0.827 (95% CI: 0.751–0.904),the p-value of DeLong test between ABCAP and AISM65 score is 0.048.

**Table 6:** Characteristics of external validation dataset

Characteristics	levels	0 (*N* = 298)	1 (*N* = 32)	*p*
Altered mental	Yes	23 (7.7%)	9 (28.1%)	0.001
Cancer	Yes	103 (34.6%)	20 (62.5%)	0.004
SBP	< 90 mmHg	130.9 ± 20.8	107.4 ± 20.6	< 0.001
		3 (1%)	7 (21.9%)	
PULSE	> 100/min	78.0 (72.0,90.0)	93.0 (77.5,109.0)	0.001
		34 (11.4%)	13 (40.6%)	
Albumin	< 30 g/L	34.3 ± 5.1	29.4 ± 6.2	< 0.001
		61 (20.5%)	17 (53.1%)	< 0.001
BUN	> 7.5 mmol/L	7.2 (4.7,10.8)	10.8 (6.8,21.2)	0.002
		138 (46.3%)	22 (68.8%)	
INR	> 1.5	1.1 (1.1,1.3)	1.4 (1.2,1.6)	< 0.001
		37 (12.8%)	12 (37.5%)	
ABCAP score		1(1,2)	2(2.5,3.25)	< 0.000
AIMS65 score		1(1,2)	2(1,3)	< 0.000

In the external validation, both the ABCAP and AIMS65 scores experienced a slight decrease in their predictive power, with the ABCAP score yielding an AUC of 0.799 (95% CI: 0.709–0.889) and the AIMS65 score achieving an AUC of 0.743 (95% CI: 0.647–0.838), the p-value of DeLong test between ABCAP and AISM65 score is 0.16. Both the ABCAP and AIMS65 scores continue to demonstrate robust risk evaluation. They maintain their potential as reliable tools for assessing severity and risk in our patient cohort.

### ABCAP score level metrics and risk stratification

Table 7 outlines the patient and death counts within the development dataset, categorized according to each score level for both the ABCAP and AIMS65 scoring systems. Notably, the distribution of scores exhibits variations across these levels, with the “0” and “1” point levels having the highest patient counts in both scoring systems. Upon analyzing cumulative counts, a clear pattern emerges wherein low scores (ranging from 0 to 2 points) and high scores (ranging from 3 to 5 points) exhibit a notable similarity for both scoring methods. However, when considering cumulative death counts and the associated ratios, we observe distinct patterns. In instances of low scores, the ABCAP system demonstrates no significant difference from the AIMS65 system. In contrast, for cases involving high scores, the ABCAP scores exhibit higher mortality ratios compared to the AIMS65 system.

Of particular interest is the “Same score patients counts” column, which reveals the count of patients who received identical scores in both the ABCAP and AIMS65 systems. Remarkably, the proportion of patients displaying the same score was consistently less than 50% across all score levels in the AIMS65 system.

**Table 7:** Score and death counts of ABCAP and AIMS65 in the development dataset

Score level	ABCAP score		AIMS65 score		Same score patients counts
	Counts	Death	Counts	Death	
0	909	2(0.2%)	0	0(NaN)	0
1	635	17(2.7%)	1338	15(1.1%)	421
2	238	31(13.0%)	396	35(8.8%)	141
Cumulative result	1782	50(2.8%)	1734	50(2.9%)	562
3	79	24(30.4%)	117	22(18.8%)	32
4	33	19(57.6%)	44	22(50.0%)	20
5	5	4(80.0%)	4	3(75.0%)	0
Cumulative result	117	47(40.2%)	165	47(28.5%)	52

Table 8 provides the cumulative mean values of the statistical metrics for each score level and the corresponding mortality rates obtained from the five imputation datasets using the ABCAP score. As score levels increase, an evident trend emerges. Sensitivity experiences a decline, while specificity follows an opposing trajectory. The Positive predictive value (PPV) exhibits an ascending pattern, whereas the negative predictive value (NPV) displays an inverse association with higher score levels.

**Table 8:** Cumulative mean of statistic metrics in total dataset use ABCAP score

Score	Sens	Spec	PPV	NPV	Death patients	All patients	Mortality%	Score level mortality%	PLR
≥ 0	1.000	0.000	0.051	NaN	97	1899	5.11	0.2	1
≥ 1	0.965	0.492	0.093	0.996	94	1010	9.27	2.7	1.898
≥ 2	0.784	0.837	0.206	0.986	76	369	20.58	13.0	4.815
≥ 3	0.476	0.961	0.397	0.972	46	116	39.69	30.4	12.232
≥ 4	0.208	0.995	0.677	0.959	20	30	67.73	57.6	40.296
5	0.041	0.999	0.797	0.951	4	5	79.67	80.0	74.309

Sens: sensitivity; Spec: specificity; PPV: positive predictive value; NPV: negative predictive value; PLR: positive likelihood ratio

In terms of mortality rates, score levels of ≥ 1 and ≥ 2 were associated with mortality rates of 9.27% and 20.58%, respectively. The positive likelihood ratio (PLR) values for these score levels are 1.898 and 4.815. For score level ≥ 3, the mortality rate further increased to 36.69%, accompanied by a PLR of 12.232. Upon reaching score levels ≥ 4 and 5, mortality rates escalated significantly to 67.73% and 79.67%. The corresponding PLR values also experience substantial increments, reaching 40.296 and 74.309, respectively.

Considering the distribution of ABCAP scores and the observed metrics at each score level, we can classify scores ranging from 0 to 2 as indicative of low risk, a score of 3 as signifying moderate risk, and scores ranging from 4 to 5 as indicative of high risk.

### ABCAP score performance in the variceal and no-variceal groups

**Fig. 4:**
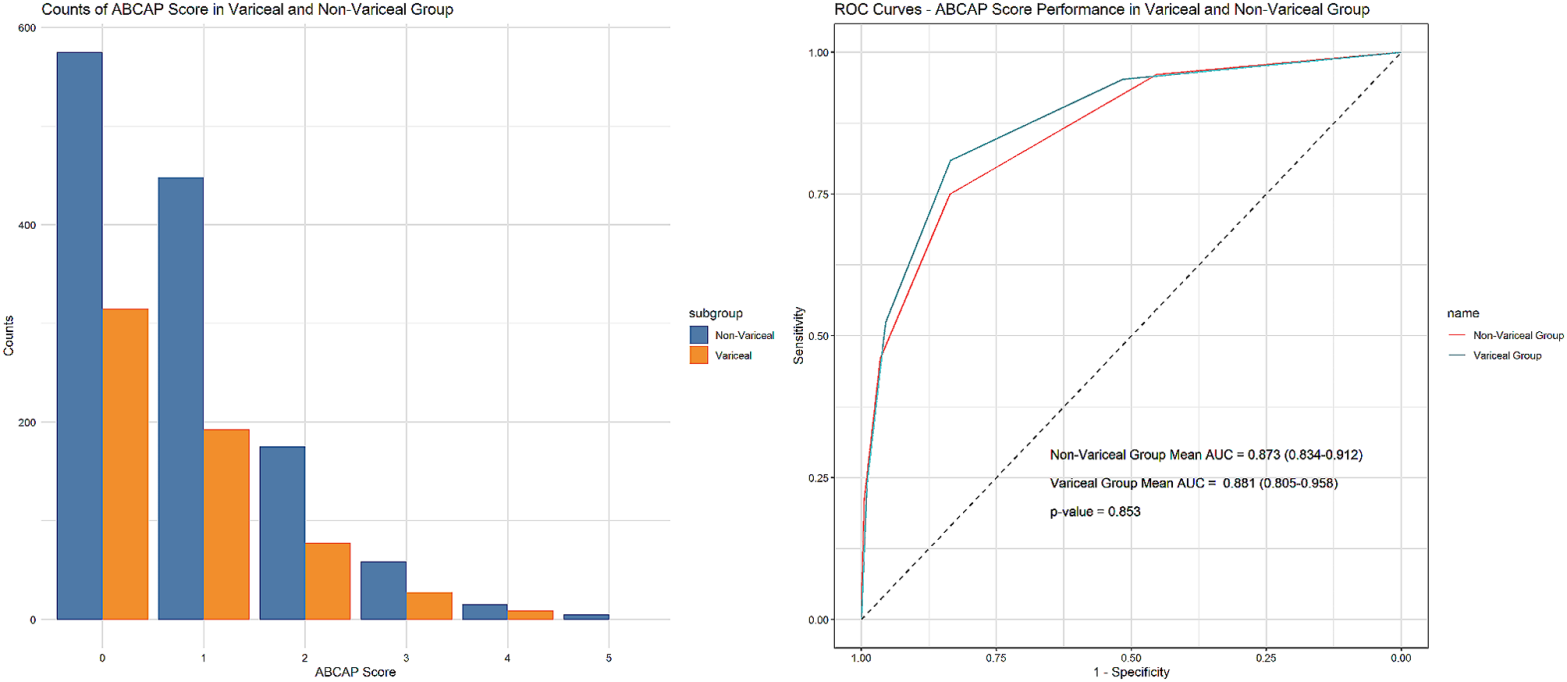
ABCAP score distribution and performance in the variceal and nonvariceal groups of the development dataset

The distribution of the ABCAP score at different score levels and its performance within the variceal (622 patients) and nonvariceal (1277 patients) groups within the Development Dataset are visualized in Fig. 4. Notably, the number of patients in the nonvariceal group was nearly double that in the variceal group. The distribution of ABCAP scores among different levels within each group correlates with the proportion of individuals in that group.

To assess the predictive performance of the ABCAP score, we utilized the AUC values for both the variceal and nonvariceal groups. In the variceal group, the calculated AUC was 0.881 (95% CI: 0.805–0.958), signifying a strong level of predictive accuracy. Similarly, within the nonvariceal group, the AUC was measured at 0.873 (95% CI: 0.834–0.912). A statistical analysis with a P value of 0.853 indicates no significant difference in the performances of the ABCAP score between these two groups.

## Discussion

Despite significant advancements in the prevention and treatment of UGIB, the prognosis for older patients remains a challenge during hospitalization. Interestingly, among the 1899 patients included in our development dataset, those who did not survive exhibited a lower rate of in-hospital endoscopy compared to the surviving group, despite the majority of patients undergoing such procedures as part of their medical care. This highlights the critical need for a simplified scoring system, designed to rapidly evaluate the prognosis of older patients, facilitating timely and appropriate medical decisions before endoscopy.

While established scoring systems such as the GBS, RS, and AIMS65 scores have undergone extensive validation and implementation for patient triage in clinical settings, it is crucial to acknowledge that the severity of various acute and chronic conditions might differ in older individuals compared to their younger counterparts [15, 16]. Thus, it becomes imperative to explore specific risk factors that address the distinct challenges encountered by this particular patient demographic.

Our study specifically targets the 30-day in-hospital mortality rate, identified at 5.1% within our dataset, compared to a broader population rate of 1.37% from 253,947 patients. This focus differs from the AIMS65, RS, and GBS scores, which generally consider overall in-hospital mortality. Concentrating on this short-term outcome allows for a direct evaluation of immediate care effectiveness and risk stratification within a crucial timeframe, offering critical insights for acute care prioritization. It also minimizes biases related to long-term follow-up, such as varying patient adherence and changes in healthcare access or treatment strategies.

To overcome the above limitations and predict short-term outcome, we developed the ABCAP score, a simplified scoring system specifically designed for older patients with UGIB.

Previous studies have primarily relied on multivariable logistic regression to identify risk factors associated with outcomes in older populations with UGIB [17]. We employed a combination of traditional methods and innovative techniques, such as StepAIC, LASSO, ENT, RFE, and Best subset selection, to facilitate the selection of key variables during the training and prediction modeling process.

Each variable selection method employed in our study—StepAIC, Best Subset selection, LASSO, ENT, and RFE—offers unique benefits and challenges. For instance, StepAIC often selects a broad set of features, which might not always be ideal, whereas Best Subset selection methodically identifies the most fitting predictor set, albeit with high computational demands and a risk of overfitting with numerous predictors. Our iterative process revealed that while StepAIC, BestSub, and RFE sometimes selected more than 10 variables, the stability of these selections varied, suggesting a risk of overfitting. Conversely, LASSO and ENT demonstrated greater consistency, selecting 6 and 7 variables respectively, showing a notable concurrence in their choices and underscoring their effectiveness in identifying a compact, predictive set of variables.

In our study, 15 variables were identified as potential predictors of in-hospital death through a rigorous selection process. Among these, Age stood out for its nuanced impact in our older population, suggesting that its predictive value might be surpassed by other determinants in individuals over 65 years old. The analysis also underscored the importance of other variables like ICH [18, 19], eGFR [20] and Peptic Ulcer Bleeding [21], with renal function indicating significant implications for UGIB outcomes and Peptic Ulcer Bleeding presenting more frequently than variceal bleeding [22]. The variability in risk associated with these conditions underscores the heterogeneity of UGIB patient outcomes, necessitating a tailored approach to risk stratification. To identify the most effective predictors, we explored various combinations of these variables, employing a comprehensive methodology to assess their collective impact.

In our analysis, we initially examined age, BMI, and SBB in continuous terms. Given their small coefficients, we opted to categorize these variables to enhance the scoring system’s interpretability and practicality. This categorization not only clarified their impact on outcomes but also aligned the model with clinical guidelines, improving real-world usability. While this transformation may reduce some information, careful cutoff selection—based on laboratory standards for BUN, Albumin, and Pulse—ensured our model’s clinical relevance and consistency with existing practices.

Ultimately, we arrived at a subset of five variables – Albumin, BUN, Cancer, Altered Mental Status, and Pulse – which were consistently selected by all five methods as well as our scoring system. This specific subset was established manually, and we are eager to evaluate its performance.

Traditional regression models have a well-established history of application and validation across various studies, leading to the development of widely used scoring systems. Notably, GBS and RS rely on logistic regression and forward stepwise techniques, respectively, while AIMS65 employs the recursive partition approach, a more recent decision tree method. Recent years, have seen the rise of machine learning techniques—RF, KNN, and SVM—enhancing prediction in areas ranging from bleeding events in valve replacement patients [23] to early Alzheimer’s detection [24] and medication adherence in chronic conditions [25]. While these advanced methods offer robust classification capabilities for categorical data, choosing the right one depends on the study’s specific needs and data characteristics. Despite their potential, machine learning approaches come with challenges, including their opaque “black box” nature and the necessity for careful parameter tuning to refine predictions.

In our study, we employed a comprehensive set of predictive modeling methods, including RF, KNN, SVM, and GLM, to conduct prediction and classification tasks. The combinations of RF, KNN, SVM, and GLM demonstrated diverse performance in predicting binary outcomes. Overall, most combinations exhibited strong performance with high accuracy and sensitivity.

Despite dedicated efforts to fine-tune the critical parameters of each machine learning approach, the results showed minimal or marginal improvements. However, it is important to highlight that generalized linear models demonstrated commendable performance and suitability in this context. This pattern led us to hypothesize that the challenges in applying machine learning methods to this specific cohort arise from its unique characteristics. Machine learning methodologies generally shine when dealing with high-dimensional, complex datasets. However, our attempts to use machine learning methods with all variables in model training yielded only marginal improvements in AUC, while complicating the prediction model considerably.

In our comprehensive comparative analysis of the three machine learning methods alongside the GLM-based combinations, with a special focus on the GLM + BestSub combination, a consistent pattern emerged. We observed that this specific combination consistently demonstrated well-balanced performance across a range of evaluation metrics. Notably, even the ABCAP score, which was manually derived from the selection of five variables, displayed slightly lower metric values in comparison to GLM + BestSub.

Our decision to develop the ABCAP score was influenced by several factors, including the need for result interpretability, data availability, domain expertise, practical ease of calculation and application.

When contrasting the ABCAP score with the GBS, RS, and AIMS65 score, there are both shared and distinct variables. For instance, BUN and Pulse, featured in the ABCAP score, are also significant factors in other scoring systems such as the GBS and RS. Additionally, Cancer is present in both the ABCAP score and the RS. The inclusion of Cancer as a predictive factor holds relevance due to its prevalence among our study population, a factor driven by the age-related increase in cancer cases and its substantial impact on prognosis [26]. Furthermore, a multicenter study on chronic diseases among older inpatients in China, utilizing our development dataset, revealed that malignancy remains the leading cause of in-hospital mortality [27].

Another significant observation within our study pertains to the dominant role of serum albumin levels, rather than HGB levels, at the time of presentation. This discovery aligns with recent research findings and the AIMS65 score, both of which emphasize the critical importance of hypoalbuminemia in forecasting mortality within the context of upper gastrointestinal bleeding and critical illness [28, 29]. Interestingly, hypoalbuminemia remains absent from the RS and GBS systems, despite its clinical relevance.

Analyzing the disparities between internal and external validation performance requires consideration of the differences in basic characteristics of the study populations. It is important to note that the AIMS65 and ABCAP scores are tailored for distinct patient groups and outcomes. Our focus on the 30-day in-hospital mortality rate diverges from AIMS65, which considers overall in-hospital mortality without a specific time frame. This divergence significantly impacts the differences in predictive performance.

During internal validation, the ABCAP score showcased superior predictive capability, reflected by its notably higher AUC value compared to the AIMS65 score. However, in external validation, while both scores experienced a reduction in performance, they remained within acceptable ranges. The ABCAP score continued to exhibit a higher AUC than the AIMS65 score, but the difference was not statistically significant, indicating comparable performance levels in external cohorts.

Given our study’s focus on individuals aged 65 and older in China, the AIMS65 score’s inherent assignment of a fixed 1-point for age possibly influences its predictive accuracy against the ABCAP, which assigns a minimum value of 0. This insight highlights the ABCAP scoring system’s robust performance and adaptability for our demographic, although it does not conclusively outperform AIMS65 in external validation.

We further delved into the patient and death counts for distinct score levels attributed to both scoring systems across the entirety of the development dataset, yielding insightful findings. Particularly noteworthy is the equilibrium observed in cumulative counts for the 1 to 2 point and 3 to 5 point categories. However, a notable divergence becomes evident when accounting for the corresponding death counts and their ratios. Within the context of our study cohort, the 3 to 5 score range of the ABCAP score exhibits a heightened ability to effectively stratify mortality, surpassing the performance of the AIMS65 score. This nuanced comparison underscores the importance of selecting an appropriate risk assessment tool that aligns with the specific characteristics of the patient population, ultimately enhancing clinical decision-making and patient care strategies.

In a cumulative analysis of the corresponding metrics across each score level in the development dataset, an upward trend in both mortality and positive likelihood ratio (PLR) was observed with increasing ABCAP scores. However, this trend was not consistently smooth. Based on the significant increase in mortality and PLR with higher scores, we were able to establish a risk stratification system. Patients with scores ranging from 0 to 2 were categorized as low risk, experiencing a mortality rate lower than 13%. A score of 3 indicated moderate risk, corresponding to a noticeable increase in mortality to 30.4%. For patients scoring 4 or 5, representing high risk, the mortality rate further escalated, ranging from 57.6 to 80%.

This risk stratification framework offers valuable guidance for healthcare providers when managing older patients with UGIB. If a patient’s calculated ABCAP score is 3 or higher, timely intervention becomes crucial due to the significantly elevated risk of mortality. Conversely, if the score is below 3, while the mortality rate remains relatively high, the prognosis is generally expected to be more favorable. This risk-based approach facilitates informed decision-making and aids in prioritizing appropriate interventions for optimal patient care.

Variceal UGIB, often linked to liver disease and esophageal or gastric varices, contrasts with nonvariceal UGIB caused by peptic ulcers or Mallory-Weiss tears. Research in UGIB commonly focuses on nonvariceal cases due to the specialized treatment variceal bleeding requires. The differentiation between variceal and nonvariceal UGIB, crucial in many studies, can be complex. For example, one investigation reported higher mortality in nonvariceal bleeding [30], while another found no significant outcome differences between the two groups [22].

In our dataset, differentiating between variceal and nonvariceal bleeding was complicated by sparse varices information, reflecting the older population’s lower endoscopy rates. Despite this, our univariable analysis indicated an association between variceal bleeding and prognosis, although it was selected as a variable by only three methods. Crucially, the ABCAP score performed consistently across both variceal and nonvariceal UGIB cases, showing no significant differences in score distribution or predictive accuracy. This underscores the ABCAP score’s utility in risk stratification for UGIB, affirming its value as a versatile prognostic tool regardless of variceal status.

### Limitation

Our study has provided valuable insights and promising results. However, certain data-related limitations need to be acknowledged. First, the relatively small sample size may limit the generalizability of our findings to a broader population. Additionally, missing data and potential biases in our retrospective study require careful consideration, even though we used imputation methods cautiously. In terms of study design, our adoption of a single-center retrospective design may introduce selection bias and limit the ability to establish causality. Conducting a multicenter prospective study would enhance the robustness of our findings.

## Conclusion

In conclusion, our developed ABCAP score, incorporating Altered Mental Status, BUN, Cancer, Albumin and Pulse as key variables, has demonstrated strong predictive performance in assessing the 30-day in-hospital mortality risk for older patients with UGIB. This score matches the predictive capacity of the AIMS65 system in internal validations and maintains consistent, albeit not statistically superior, performance in external validations. Notably, at the score levels of 3 to 5, ABCAP demonstrates a unique advantage in identifying high-risk patients more effectively than AIMS65, underscoring its potential for more nuanced risk stratification. Importantly, the ABCAP score effectively stratifies mortality risk in both variceal and nonvariceal bleeding cases. Nonetheless, to establish its wider applicability and generalizability, further validation studies across diverse healthcare settings and patient populations are imperative. With its potential to provide valuable risk assessment insights, the ABCAP score stands as a promising tool to guide clinicians in making well-informed decisions and prioritizing appropriate interventions during the acute care phase for older UGIB patients.

### Electronic supplementary material

Below is the link to the electronic supplementary material.

Supplementary Material 1

## Data Availability

We regret to inform that the original data used in this study cannot be made publicly available due to restrictions imposed by our affiliated institution. The data contain sensitive patient information, and our institution has not granted approval for its public release. However, interested researchers may request access to the de-identified data, subject to approval from our institutional ethics committee. Requests for data access can be directed to xuezaiyao@163.com.

## References

[1] Hearnshaw SA, Logan RF, Lowe D, Travis SP, Murphy MF, Palmer KR (2011). Acute upper gastrointestinal bleeding in the UK: patient characteristics, diagnoses and outcomes in the 2007 UK audit. Gut.

[2] Peery AF, Crockett SD, Murphy CC, Lund JL, Dellon ES, Williams JL, Jensen ET, Shaheen NJ, Barritt AS, Lieber SR (2019). Burden and cost of gastrointestinal, liver, and pancreatic diseases in the United States: Update 2018. Gastroenterology.

[3] Rockall TA, Logan RF, Devlin HB, Northfield TC (1996). Risk assessment after acute upper gastrointestinal haemorrhage. Gut.

[4] Zhong M, Chen WJ, Lu XY, Qian J, Zhu CQ (2016). Comparison of three scoring systems in predicting clinical outcomes in patients with acute upper gastrointestinal bleeding: a prospective observational study. J Dig Dis.

[5] Blatchford O, Murray WR, Blatchford M (2000). A risk score to predict need for treatment for upper-gastrointestinal haemorrhage. Lancet.

[6] Saltzman JR, Tabak YP, Hyett BH, Sun X, Travis AC, Johannes RS (2011). A simple risk score accurately predicts in-hospital mortality, length of stay, and cost in acute upper GI bleeding. Gastrointest Endosc.

[7] Wang H, Chen H (2022). Aging in China: challenges and opportunities. China CDC Wkly.

[8] Na L, Carballo KV, Pauphilet J, Haddad-Sisakht A, Kombert D, Boisjoli-Langlois M, Castiglione A, Khalifa M, Hebbal P, Stein B et al. Patient Outcome Predictions Improve Operations at a Large Hospital Network. *arxiv:230515629[csLG,csAI]*.

[9] Laine L, Barkun AN, Saltzman JR, Martel M, Leontiadis GI (2021). ACG Clinical Guideline: Upper Gastrointestinal and Ulcer bleeding. Am J Gastroenterol.

[10] Park SW, Song YW, Tak DH, Ahn BM, Kang SH, Moon HS, Sung JK, Jeong HY (2015). The AIMS65 score is a useful predictor of mortality in patients with Nonvariceal Upper gastrointestinal bleeding: urgent endoscopy in patients with high AIMS65 scores. Clin Endosc.

[11] Neumann JT, Thao LTP, Callander E, Chowdhury E, Williamson JD, Nelson MR, Donnan G, Woods RL, Reid CM, Poppe KK (2022). Cardiovascular risk prediction in healthy older people. Geroscience.

[12] Bloom AS, Suchindran S, Steinbrink J, McClain MT (2019). Utility of predictive tools for risk stratification of elderly individuals with all-cause acute respiratory infection. Infection.

[13] Charlson ME, Pompei P, Ales KL, MacKenzie CR (1987). A new method of classifying prognostic comorbidity in longitudinal studies: development and validation. J Chronic Dis.

[14] Quan H, Li B, Couris CM, Fushimi K, Graham P, Hider P, Januel JM, Sundararajan V (2011). Updating and validating the Charlson comorbidity index and score for risk adjustment in hospital discharge abstracts using data from 6 countries. Am J Epidemiol.

[15] Oakland K, Guy R, Uberoi R, Hogg R, Mortensen N, Murphy MF, Jairath V, Collaborative UKLGB (2018). Acute lower GI bleeding in the UK: patient characteristics, interventions and outcomes in the first nationwide audit. Gut.

[16] Dumic I, Nordin T, Jecmenica M, Stojkovic Lalosevic M, Milosavljevic T, Milovanovic T. Gastrointestinal Tract Disorders in Older Age. *Can J Gastroenterol Hepatol* 2019, 2019:6757524. 10.1155/2019/6757524 PMC6354172 30792972

[17] Redondo-Cerezo E, Ortega-Suazo EJ, Vadillo-Calles F, Valverde-Lopez F, Martinez-Cara JG, Jimenez-Rosales R (2021). Upper gastrointestinal bleeding in patients 80 years old and over. A comparison with younger patients and risk factors analysis for in-hospital and delayed mortality. Int J Clin Pract.

[18] Qiu W, Liu C, Ye J, Wang G, Yang F, Pan Z, Hu W, Gao H (2023). Age-to-Glasgow Coma Scale score ratio predicts gastrointestinal bleeding in patients with primary intracerebral hemorrhage. Front Neurol.

[19] Wei J, Jiang R, Li L, Kang D, Gao G, You C, Zhang J, Gao L, Huang Q, Luo D (2019). Stress-related upper gastrointestinal bleeding in adult neurocritical care patients: a Chinese multicenter, retrospective study. Curr Med Res Opin.

[20] Bai Z, Primignani M, Guo X, Zheng K, Li H, Qi X (2019). Incidence and mortality of renal dysfunction in cirrhotic patients with acute gastrointestinal bleeding: a systematic review and meta-analysis. Expert Rev Gastroenterol Hepatol.

[21] Leontiadis GI, Molloy-Bland M, Moayyedi P, Howden CW (2013). Effect of comorbidity on mortality in patients with peptic ulcer bleeding: systematic review and meta-analysis. Am J Gastroenterol.

[22] Tandon P, Bishay K, Fisher S, Yelle D, Carrigan I, Wooller K, Kelly E (2018). Comparison of clinical outcomes between variceal and non-variceal gastrointestinal bleeding in patients with cirrhosis. J Gastroenterol Hepatol.

[23] Kim J, Jang I (2021). Predictors of bleeding event among elderly patients with mechanical valve replacement using random forest model: a retrospective study. Med (Baltim).

[24] Elgammal YM, Zahran MA, Abdelsalam MM (2022). A new strategy for the early detection of alzheimer disease stages using multifractal geometry analysis based on K-Nearest neighbor algorithm. Sci Rep.

[25] Lee SK, Kang BY, Kim HG, Son YJ (2013). Predictors of medication adherence in elderly patients with chronic diseases using support vector machine models. Healthc Inf Res.

[26] Smith BD, Smith GL, Hurria A, Hortobagyi GN, Buchholz TA (2009). Future of cancer incidence in the United States: burdens upon an aging, changing nation. J Clin Oncol.

[27] Feng C, Yabin W, Wanguo X, Hongbin L, Xin L, Tianzhi L, et al. Clinical multi-centers report of chronic diseases elderly inpatients in China. Chin J Multiple Organ Diseases in the Elderly. 2018;17(11):801–8.

[28] Freire AX, Bridges L, Umpierrez GE, Kuhl D, Kitabchi AE (2005). Admission hyperglycemia and other risk factors as predictors of hospital mortality in a medical ICU population. Chest.

[29] Tung CF, Chow WK, Chang CS, Peng YC, Hu WH (2007). The prevalence and significance of hypoalbuminemia in non-variceal upper gastrointestinal bleeding. Hepatogastroenterology.

[30] Ratiu I, Lupusoru R, Popescu A, Sporea I, Goldis A, Danila M, Miutescu B, Moga T, Barbulescu A, Sirli R (2022). Acute gastrointestinal bleeding: a comparison between variceal and nonvariceal gastrointestinal bleeding. Med (Baltim).

