# Supplementary material for: Prediction of 30-day in-hospital mortality in older UGIB patients using a simplified risk score and comparison with AIMS65 score
Source: BMC Geriatr. 2024 Jun 20;24:534. doi: 10.1186/s12877-024-04971-w (PMC11188522; doi:10.1186/s12877-024-04971-w)
Supplement: Supplementary file 1 — Supplementary Material 1 [file 12877_2024_4971_MOESM1_ESM.docx]

1.ICD-10 related in data collection

| Upper Gastrointestinal Bleeding | K20.06、K22.601、K22.803、K25.001、K25.002、K25.201、K25.401、K25.402、K25.403、K26.001、K26.002、K26.201、K26.401、K26.402、K26.403、K26.601、K27.001、K27.002、K27.401、K27.402、K27.403、K27.404、K29.001、K29.003、K70.303、K71.702、K74.303、K74.607、K74.608、K92.001、K92.204、K92.206 |
| --- | --- |
| Hypertension | I10-13, I15 |
| Atrial Fibrillation | I48 |
| Charlson Comorbidity Index |  |
| Myocardial Infarction | I21, I22, I25.2 |
| Congestion Heart Failure | I50.0-I50.9, I11.0, I13.0, I13.2 |
| Peripheral vascular diseases | I70、K55 |
| Cerebrovascular disease | G45，G46，I60-69 |
| Dementia | F01-06、G30、G31、R54 |
| Chronic obstructive pulmonary disease | J44 |
| Connective tissue diaease | M05-06，M315，M32-34、M351、M353、M360 |
| Peptic ulcer | K25-28 |
| Mild liver disease | B15-B19，K71，K3，K75 |
| Moderate&Severe liver disease | K72-74，K76-77 |
| Diabetes without complications | E10.0、E10.9、E11.0-1、E11.9、E13.9、E14.1、E14.9 |
| Diabetes with complications | E10.2-10.7、E11.2-11.7、E13.2-13.5、E14.2-14.6 |
| Hemiplegia | G80-83 |
| Moderate&Severe kidney disease | N18 |
| Localized Cancer | C00-69，C70-76，C80 |
| Leukemia | C91-96，D46 |
| Lymphoma | C81-90 |
| Metastatic | C77-79，M8+“meta” |
| AIDS | B20-24 |

2.Imputation iterations Iterations of Variables with missing value compare with corresponding variable before imputation(t-test and nonparametric tests)

| Imputation iterations | p_value |
| --- | --- |
| No.1 Imputation |  |
| SBP | 0.987 |
| PULSE | 0.992 |
| Platelet | 0.436 |
| HGB | 0.750 |
| Albumin | 0.625 |
| BUN | 0.966 |
| INR | 0.748 |
| EGFR | 0.463 |
| No.2 Imputation |  |
| SBP | 0.989 |
| PULSE | 0.989 |
| Platelet | 0.427 |
| HGB | 0.732 |
| Albumin | 0.654 |
| BUN | 0.958 |
| INR | 0.692 |
| EGFR | 0.375 |
| No.3 Imputation |  |
| SBP | 0.997 |
| PULSE | 0.988 |
| Platelet | 0.386 |
| HGB | 0.717 |
| Albumin | 0.675 |
| BUN | 0.968 |
| INR | 0.696 |
| EGFR | 0.392 |
| No.4 Imputation |  |
| SBP | 0.994 |
| PULSE | 0.990 |
| Platelet | 0.361 |
| HGB | 0.730 |
| Albumin | 0.662 |
| BUN | 0.876 |
| INR | 0.697 |
| EGFR | 0.491 |
| No.5 Imputation |  |
| SBP | 0.997 |
| PULSE | 0.986 |
| Platelet | 0.525 |
| HGB | 0.850 |
| Albumin | 0.748 |
| BUN | 0.982 |
| INR | 0.706 |
| EGFR | 0.391 |
